# Stroke incidence and case fatality: a 9-year prospective population-based study in an elderly population of Bagheria, Italy

**DOI:** 10.1007/s10072-020-04830-7

**Published:** 2020-10-19

**Authors:** Valentina Arnao, Giuseppe Salemi, Salvatore Scondotto, Nicola Casuccio, Marianna Riolo, Marco D’Amelio, Paolo Ragonese, Paolo Aridon

**Affiliations:** 1 Department of Biomedicine, Neuroscience and Advanced Diagnostics (BiND), University of Palermo, Via Gaetano la Loggia n.1, 90129 Palermo, Italy; 2 UO Neurologia e Stroke Unit, A. R. N. A. S. Ospedali Civico Di Cristina Benfratelli, Palermo, Italy; 3 Sorveglianza Ed Epidemiologia Valutativa-Regione Sicilia, Palermo, Italy; 4 Azienda sanitaria Provinciale di Palermo -UOC di sanità pubblica, epidemiologia e medicina preventiva, Palermo, Italy; 5 Ospedale Santa Croce di Moncalieri - Asl TO5, Moncalieri, Torino, Italy

**Keywords:** Epidemiology, Prevalence, Incidence, 28-day case fatality rate, Stroke

## Abstract

**Background:**

The incidence of stroke in high-income countries has been on the decline; however, few epidemiological surveys have been conducted in recent years to specifically estimate the incidence along with outcome of stroke, in Italy. This study aimed to examine the incidence and case fatality rates of stroke in an elderly Italian population.

**Methods:**

A cohort of 2200 people > 65 years was randomly stratified from the total elderly population of Bagheria, Italy. A 9-year prospective population-based study was performed (19,800 person/years).

**Results:**

We identified 112 first-ever strokes, 53 females and 59 males: 82 (73.1%) ischemic, 13(11.6%) intracerebral haemorrhages, 6 (5.35%) subarachnoid haemorrhages, while 11(9.8%) were classified as undetermined strokes. The crude overall annual incidence was 5.65 per 1000 (95%CI: 4.61 to 6.70) for first-ever stroke. The overall crude incidence rates were 4.74 per 1000 (5.08 for males and 4.46 for females) for ischemic stroke, 0.65 (0.99 for males and 0.37 for females) for intracerebral haemorrhage, and 0.03 for subarachnoid haemorrhage. The incidence rate for first-ever stroke was 5.4 per 1000 (95% CI: 5.36 to 5.45) after adjustment for the 2015 World population and 5.56 (95% CI: 5.52 to 5.61), compared to the 2015 European population. Overall case fatality rates for first-ever stroke was 8.19% at 28 days and 24.1% at 1 year**.**

**Conclusion:**

Our study shows that in the elderly population investigated, stroke incidence and case fatality rates resulted being lower, compared to those from Italian and most European populations. Similar to previous studies, these rates increased linearly with age and were higher in males.

## Introduction

Stroke remains a major health problem worldwide. In high-income countries, it represents the second cause of death and the third leading cause of disability [1], whereas low- and middle-income countries have been experiencing an increased incidence over the last years [2].

Stroke is a complex disease resulting from the interaction of numerous environmental and genetic factors. A detailed investigation of lifestyle, diet, and genetic predisposition cannot be separated from epidemiological rates, especially incidence and mortality. In fact, changes in trends for both incidence and mortality rates are important in identifying the effects of changes in modifiable risk factors, screening factors, and prevention on stroke trends. Our objective was to assess for the incidence along with the 28-day and 1-year case fatality rates of first-ever stroke in a Sicilian population using data from a population-based survey of elderly participants.

## Methods

In a previous paper, we described a survey (Prevalence day September 30, 2006) that was carried out in the municipality of Bagheria, a town nearby Palermo. Briefly, a cohort of 2200 people over 65 years of age, representing 25% of the whole population, was randomly stratified [3]. A semi-structured questionnaire was administered to each subject and, as a second level, a neurologist evaluated people reporting neurological symptoms. Nine years later, the clinical data for the whole cohort were obtained from a local health institution and from the general practitioners (GPs) of the study area. In addition, we examined the Hospital Discharge Diagnosis Data Bank, a computerized system that collects the diagnosis of each hospitalized patient of the study area. We primarily looked for patients having a discharge diagnosis including codes 430 to 438, according to the International Classification of Disease, 9th revision. Mortality, cause of mortality data, and death certificates were obtained from local institutions. In particular, the local Health Registry of the Sanitary District (ASP 6) provided the dates along with information on dates and causes of death.

According to the WHO criteria, stroke was considered “rapidly developing clinical symptoms or signs of focal, and at times global, loss of cerebral function, with symptoms lasting more than 24 hours or leading to death, with no apparent cause other than that of vascular origin” [4]. In this follow-up study, we included only cases of first-ever stroke, considering it as an event occurring for the first time in a patient’s lifetime. Discharge diagnosis codes, clinical data obtained from either hospitalization or GPs permitted us to categorize ischemic stroke (IS), intracerebral haemorrhage (ICH), subarachnoid haemorrhage (SAH), or undetermined stroke (UND). Patients with transient ischemic attacks (TIA) or recurrent stroke were not included in the analysis of “first-ever group”.

The subjects were re-evaluated at the end of follow-up period (December 31, 2015). Crude-, age-, and sex-specific incidence rates with 95% confidence intervals were calculated. Person/years were obtained multiplying the mean of the population by nine (length of the follow-up period). All rates were adjusted to the 2015 World, European, and Italian population [5]. The 28-day and 1-year case fatality rates were defined as the number of deaths due to stroke (or stroke type) divided by the total number of cases of stroke (or stroke type). The local Ethical and Scientific Committee (Palermo 1, v. 5/2015, May 13, 2015) approved this study.

## Results

We identified 176 patients with cerebrovascular disease during the follow-up period. Eighty-four (47.3%) were female with a mean age of 76 (range 65 to 92) and ninety-two (52.7%) were male with a mean age of 75 (65 to 87). Of these 176, 112 (63.6%) were first-ever strokes (patients’ characteristics have been summarized in Table 1), 59 were male with a mean age of 75 years (range 65 to 87), and 53 were female (mean age 76 years); 53 (29.5%) were transient ischemic attacks and 11 were described as code 438 (late effects of cerebrovascular disease). Almost all patients underwent neuroimaging study. Hence, one hundred and twelve cases were included in the study: 82 were classified as ischemic stroke (73.1%), 13 intracerebral haemorrhages (11.6%), and 6 subarachnoid haemorrhages (5.35%); 11 were classified as undetermined strokes (9.8%) because they did not undergo a neuroimaging test.

**Table 1:** Characteristics of patients with first-ever stroke in Bagheria, according to the type of index stroke

	Total (= 112)	IS (= 82)	ICH (= 13)	SAH (= 6)	Unknown (= 11)
Male (%)	59 (52.6)	40 (48.7)	9 (69.2)	4 (66.6)	6 (54.5)
Age (SD)	79.2 (± 5.9)	79.0 (± 5.6)	81.1 (± 7.3)	76.4 (± 5.2)	79.5 (± 6.6)
Education (%)					
Elementary or below	92 (82.1)	66 (80.4)	11 (84.6)	5 (83.3)	10 (90.9)
Middle school	11 (9.8)	9 (10.9)	1 (7.6)	1 (16.7)	0
High school or above	9 (8.1)	7 (8.5)	1 (7.6)	0	1 (9.1)
Smoking status (%)					
Never	53 (47.3)	45 (54.8)	4 (30.8)	1 (16.7)	3 (27.2)
Past smoker	11 (9.8)	6 (7.3)	2 (15.4)	1 (16.7)	2 (18.1)
Current smoker	41 (36.6)	27 (32.9)	5 (38.5)	4 (66.7)	5 (45.4)
Unknown	7 (6.3)	4 (4.8)	2 (15.4)	0	1 (9.1)
Drinking status (%)					
Never	62 (55.4)	50 (60.9)	5 (38.5)	2 (33.3)	5 (45.4)
Past drinker	3 (2.7)	1 (1.2)	1 (7.7)	0	1 (9.1)
Current drinker	40 (35.7)	27 (32.9)	5 (38.5)	4 (66.7)	4 (36.3)
Unknown	7 (6.3)	4 (4.8)	2 (15.4)	0	1 (9.1)
BMI (kg/m^2^)					
< 18.5	0	0	0	0	0
18.6–24.9	27 (24.1)	22 (26.8)	5 (38.5)	0	0
25–29.9	45 (40.1)	29 (35.3)	3 (23.1)	4 (66.7)	9 (81.8)
≥ 30	20 (17.8)	15 (18.2)	3 (23.1)	1 (16.7)	1 (9.1)
Unknown	20 (17.8)	16 (19.5)	2 (15.4)	1 (16.7)	1 (9.1)
Hypertension (%)					
Yes	65 (58)	47 (57.3)	7 (53.8)	4 (66.7)	7 (63.6)
No	39 (34.8)	30 (36.5)	4 (30.8)	2 (33.3)	3 (27.2)
Unknown	8 (7.1)	5 (6.2)	2 (15.4)	0	1 (9.1)
Diabetes (%)					
Yes	27 (24.1)	22 (26.8)	2 (15.4)	1 (16.7)	2 (18.1)
No	78 (69.6)	56 (68.2)	9 (69.2)	5 (83.3)	8 (72.7)
Unknown	7 (6.3)	4 (4.9)	2(15.4)	0	1 (9.1)
Dyslipidemia (%)					
Yes	22 (19.6)	16 (19.5)	2 (15.4)	0	4 (36.3)
No	83 (74.1)	62 (75.6)	9 (69.2)	6 (100)	6 (54.5)
Unknown	7 (6.2)	4 (4.8)	2 (15.4)	0	1 (9.1)
TIA (%)					
Yes	6 (5.4)	5 (6.1)	1 (7.7)	0	0
No	91 (81.3)	63 (76.8)	12 (92.3)	6 (100)	10 (90.9)
Unknown	15 (13.4)	14 (17.0)	0	0	1(9.1)
Cardiovascular diseases (%)					
Yes	36 (32.1)	23 (28.0)	5 (38.5)	2 (33.3)	6 (54.5)
No	69 (61.6)	55 (67.0)	6 (46.2)	4 (66.7)	4 (36.3)
Unknown	7 (6.3)	4 (4.9)	2 (15.4)	0	1 (9.1)
Medications before admission (%)					
Antihypertensive med.	46 (41.1)	35 (42.6)	3 (23.1)	2 (33.3)	6(54.5)
Antithrombotic med.	3 (2.7)	3 (3.2)	0	0	0
Diabetes med.	25 (22.3)	20 (24.3)	2 (15.4)	1 (16.7)	2 (18.1)
Lipid-lowing med.	18 (16.1)	14 (17.1)	1 (7.7)	0	3 (27.2)

*BMI*, body mass index; *ICH*, intracerebral haemorrhage; *IS*, ischemic stroke; *SAH*, subarachnoid haemorrhage; *Med*, medicines. Unless indicated otherwise, data are given as *n* (%) or the mean ± SD

The overall crude incidence rate for first-ever stroke (*N* = 112) was 5.65 per 1000 (95%CI: 4.61 to 6.70), showing higher rates in males 6.52 (95% CI: 4.86 to 8.18), compared to females (4.92 per 1000; 95% CI: 3.60 to 6.25). Therefore, the incidence rate adjusted to the 2015 World population was 5.40 (95%CI: 5.22 to 5.58): 6.09 (95%CI: 5.79 to 6.38) for males and 4.67 (95%CI: 4.43 to 4.90) for females.

The incidence rates adjusted to the European and Italian population were 5.56 (95%CI: 5.16 to 5.96) and 5.62 (95%CI: 4.35 to 6.89), respectively (Table 2).

**Table 2:** Age- and sex-specific incidence rates of cerebrovascular diseases and first-ever stroke per 1000 person-years in Bagheria, an elderly community: Incidence of first-ever stroke

Age group	Males			Females			All		
	P/y	Cases	Rate	P/y	Cases	Rate	P/y	Cases	Rate
65–69	2043	9	4.40	2394	8	3.34	4437	17	3.83
70–74	2898	17	5.86	2925	16	5.47	5823	33	5.66
75–79	2142	16	7.47	2664	12	4.50	4806	28	5.82
80–84	1341	14	10.44	1611	11	6.82	2952	25	8.46
85+	621	3	4.83	1161	6	5.16	1782	9	5.05
All ages	9045	59	6.52	10,755	53	4.92	19,800	112	5.65
			*6.09 #6.29 °6.34			*4.67 #4.79 °4.85			*5.40 #5.56 °5.62

*Adjusted to the world population; ^#^Adjusted to the European population; °Adjusted to the Italian population

**Fig. 1:**
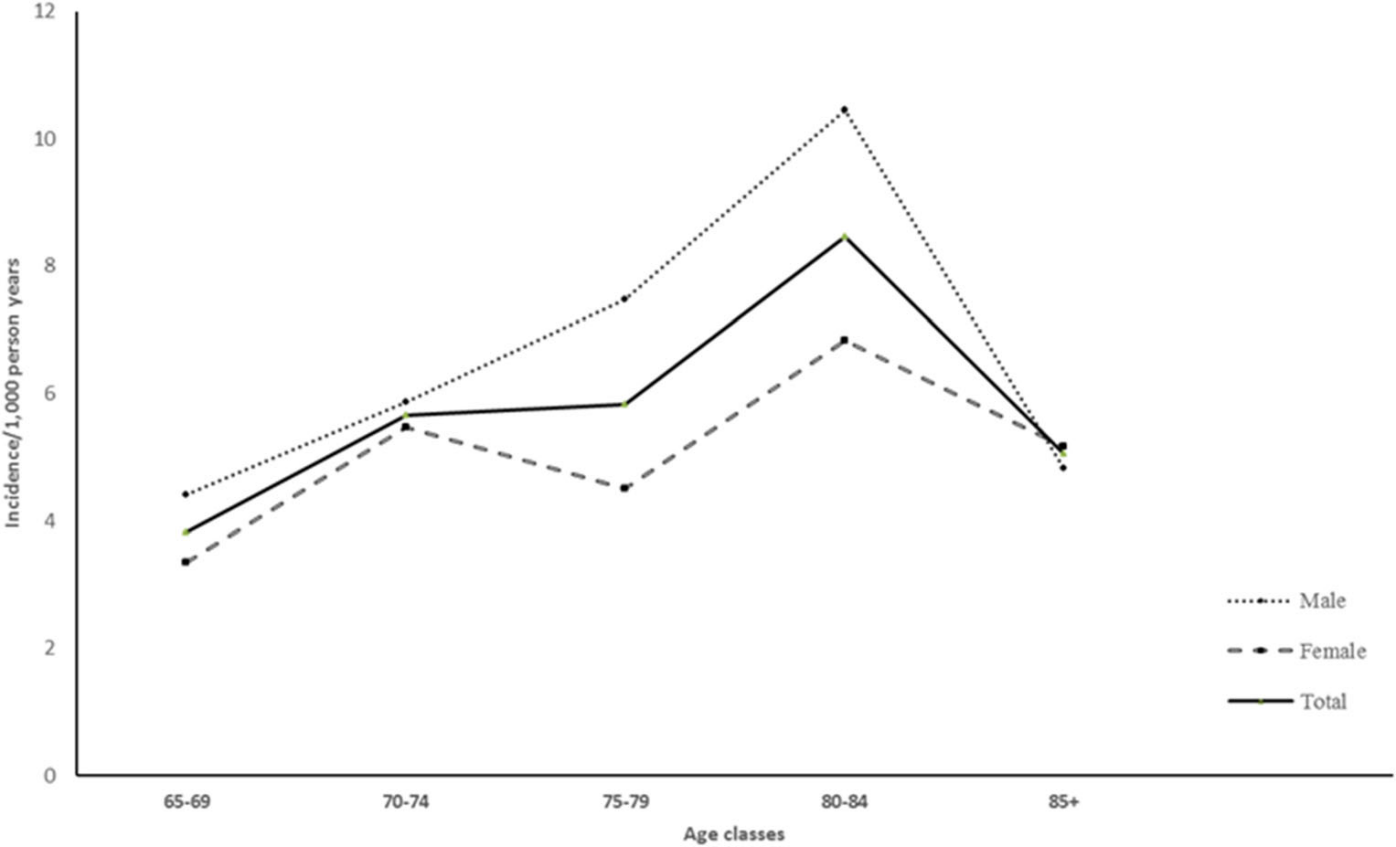
Age- and sex-specific incidence rates of first-ever stroke in Bagheria

Age-specific incidence rates of first-ever stroke increased with advancing age in both sexes, with a peak of 8.46 per 1000 in narrow 5-year age classes after the age of 80 years, 10.44 per 1000 for males and 6.82 per 1000 for females (Fig. 1 and Table 2).

Regarding the different subtypes of stroke, the overall crude incidence rates were 4.14 (4.42 for males and 3.90 for females) per 1000 for ischemic stroke, 0.65 (0.99 for males and 0.37 for females) for intracerebral haemorrhage and 0.30 (0.44 for males and 0.18 for females) for subarachnoid haemorrhage (Tables 3, 4, and 5). Once again, these values rose with age and reached a peak between 80 and 84 years of age for ischemic, and 75–79 years and over 85 years, respectively, for males and females for intracerebral haemorrhage.

**Table 3:** Age- and sex-specific incidence rates of cerebrovascular diseases and first-ever stroke per 1000 person-years in Bagheria, an elderly community: Incidence of ischemic stroke

Age Group	Males			Females			All		
	P/y	Cases	Rate	P/y	Cases	Rate	P/y	Cases	Rate
65–69	2043	7	3.42	2394	7	2.92	4437	14	3.15
70–74	2898	12	4.14	2925	12	4.10	5823	24	4.127
75–79	2142	10	4.66	2664	10	3.75	4806	20	4.16
80–84	1341	9	6.71	1611	9	5.58	2952	18	6.09
85+	621	2	3.22	1161	4	3.44	1782	6	3.36
All ages	9045	40	4.42	10,755	42	3.90	19,800	82	4.14

**Table 4:** Age- and sex-specific incidence rates of cerebrovascular diseases and first-ever stroke per 1000 person-years in Bagheria, an elderly community: Incidence of intracerebral haemorrhage

Age group	Males			Females			All		
	P/y	Cases	Rate	P/y	Cases	Rate	P/y	Cases	Rate
65–69	2043	1	0.48	2394	0	0.0	4437	1	0.22
70–74	2898	2	0.69	2925	2	0.68	5823	4	0.68
75–79	2142	4	1.86	2664	0	0.0	4806	4	0.83
80–84	1341	2	1.49	1611	0	0.0	2952	2	0.67
85+	621	0	0	1161	2	1.72	1782	2	1.12
All ages	9045	9	0.99	10,755	4	0.37	19,800	13	0.65

**Table 5:** Age- and sex-specific incidence rates of cerebrovascular diseases and first-ever stroke per 1000 person-years in Bagheria, an elderly community: Incidence of subarachnoid haemorrhage

Age group	Males			Females			All		
	P/y	Cases	Rate	P/y	Cases	Rate	P/y	Cases	Rate
65–69	2043	1	0.48	2394	1	0.41	4437	2	0.45
70–74	2898	2	0.69	2925	0	0	5823	2	0.34
75–79	2142	0	0	2664	1	0.37	4806	1	0.20
80–84	1341	1	0.74	1611	0	0.0	2952	1	0.33
85+	621	0	0	1161	0	0	1782	0	0
All ages	9045	4	0.44	10,755	2	0.18	19,800	6	0.30

The overall mean age of death was 82.6 years (81.3 for males and 84.2 for females). The higher mortality (54%) was for intracerebral haemorrhage (7 /13 patients) compared to subarachnoid haemorrhage (50%; 3/6 patients) and ischemic stroke (44%; 41/93 patients). The overall case fatality rates at 28 and 30 days were equal at 8.9% (10/112 patients): 10.3% for males (6/58 patients, mean age of death 78 years) and 7.4% for females (4/54 patients, mean age of death 83 years). The overall 1-year case fatality rate was 24.1 (27/112 patients; mean age of death 81): 27.5% for males (16/58 patients; mean age at death 80 years) and 20.3% for females (11/54 patients, mean age at death 82 years).

## Discussion

Over the last years, several studies on the incidence and case fatality of stroke have been conducted, but it must be considered that a comparison of results cannot always be possible, due to differing study designs and types of population used. In this population-based study, we report on the incidence and case fatality rates for first-ever stroke, in an elderly population from a Sicilian municipality.

We report an incidence rate lower than those obtained by other studies using European populations. Similar to previous studies, incidence increased linearly with age and was higher in males than in females. It is worth noting that Manobianca et al. [6] reported, for the age groups 65–74, 75–84, and over 85, incidence rates of 3.5, 11, and 21.4 per 1000, respectively, while Musolino et al. [7] for the same age groups reported rates of 7.46, 9.65, and 12.18, respectively. In our study, the lower incidence confirms recent scientific evidence of a decline in stroke incidence in Italy [8], reported by a 10-year population-based study carried out in the Lombardy Region in Northern Italy. Moreover, these decreased rates may also have been attributable to protective factors related to varied cultures and lifestyles in the different study areas. Thus, adherence to the Mediterranean diet, a better control of cardiovascular risk factors, especially smoking habits and alcohol consumption, could make our results unsurprising.

In our study, higher incidence rates were found for males. The lower frequency of smoking and alcohol consumption in older females, in addition to a lack of exposure to environmental factors (e.g. agricultural chemicals, herbicide), could partially explain these results.

The distribution of different stroke subtypes was similar to those reported in other studies, but our incidence was proportionally lower.

In previous European studies, 30-day case fatality ranged from 18.1% in Acquaviva Casamassima [9] to 20.3 in Trasimeno [10] or 23.7 in Vibo Valentia (28-day case fatality) [11]. Likewise, the 1-year case fatality varied from 39.1 on the Aeolian Island [12] to 40.2 in Vibo Valentia [11]. In our population, the 30-day and 1-year mortality rates were the lowest reported in Italy [13, 14]. Despite this, it could be easy to hypothesise that these differences may be due to an improved global health; however, we must consider that this is the first study to be conducted on this area in 30 years and to compare the results from different geographical areas could lead to unreliable conclusions. Furthermore, we cannot consider our data as the result of an improvement in standard of care provided by stroke unit care, as the closest stroke units to the study area were at least 20 km away, and an efficient stroke network for expedient transfer is lacking. It must be stated here that none of the patients included in this study received thrombolysis and/or thrombectomy. Moreover, we can hypothesise that the observed better survival in the elderly population may be due to a real improvement in the quality of medical care for stroke patients at Sicilian hospitals, or to an underdiagnosis of severe stroke, as these patients probably never sought out hospital care or did not have the possibility to reach an emergency care area. In addition, general practitioners filling out death certificates could not include stroke as the cause of death.

Despite a 9-year-period of observation, the magnitude of the sample size could be considered a limitation. On the contrary, the present study has several strengths. First, it was conducted in a well-defined geographical district in Sicily, including all the multiple sources available to ascertain and characterize stroke occurrence. Second, the clinical definition, according to the WHO criteria, and the choice to include in the analyses only the first-ever strokes, considering the information on stroke subtypes, permitted for the comparison with other studies.

In conclusion, the findings of this study suggest that this Sicilian population, as the Southern Italian population and other industrialized European countries, needs to be monitored for incidence and case fatality due to cerebrovascular disease, with a particular attention paid toward identifying stroke subtype, mortality, and any risk factors that allow for the development of effective public health approaches aimed at preventing stroke.

## Data Availability

The data that support the findings of this study are available from the corresponding author upon reasonable request.
